# The Role of the Ketogenic Diet in Modulating Biochemical Pathophysiology in Psychiatric and Neurodegenerative Disorders

**DOI:** 10.3390/ijms27114932

**Published:** 2026-05-29

**Authors:** Yoo Been Chang, James D. Baleja

**Affiliations:** 1 Master’s Program in Biomedical Sciences, Tufts University School of Medicine, 136 Harrison Avenue, Boston, MA 02111, USA; 2 Departments of Medical Education and Developmental, Molecular, and Chemical Biology, Tufts University School of Medicine, 136 Harrison Avenue, Boston, MA 02111, USA

**Keywords:** ketogenic diet, mitochondrial dysfunction, oxidative stress, brain energy metabolism, neurotransmitter dysregulation, schizophrenia, bipolar disorder, Alzheimer’s disease

## Abstract

The ketogenic diet, a high-fat and low-carbohydrate diet, has potential therapeutic effects on various neurological and psychiatric disorders. The diet shifts the body’s energy production in the form of adenosine triphosphate from using glucose to fats. The increased fatty acid β-oxidation results in the production of ketone bodies. This metabolic adaptation changes cellular bioenergetics, especially in the brain, which is highly reliant on energy metabolism. Schizophrenia, a psychotic disorder, and bipolar disorder, a mood disorder, are distinct psychiatric illnesses that can both involve disturbances in mood, cognition, and perception. These disturbances differ in prominence and clinical significance between the two conditions. Although the underlying mechanisms behind each disorder vary, they share some common pathophysiology, such as imbalances in the neurotransmitter system, mitochondrial dysfunction, and oxidative stress. Alzheimer’s disease, a neurodegenerative disorder marked by progressive cognitive decline, shares similar cellular disruptions, along with additional pathological features such as neuroinflammation and neuronal death. Recent studies suggest that the ketogenic diet may exert therapeutic effects by modulating underlying biochemical pathways. Its ability to reduce oxidative stress, improve mitochondrial function, and stabilize neurotransmitter balance may help alleviate symptoms and potentially slow disease progression.

## 1. Introduction

The ketogenic diet (KD) is a high-fat, low-carbohydrate eating regimen that has gained significant attention for its therapeutic potential in treating various medical conditions. Particularly, it has recently gained attention for its potential benefit in neurological disorders. Ketogenic diets are typically characterized by a carbohydrate intake of less than 50 g per day; however, in many individuals, intake must be reduced to approximately 20–30 g per day to achieve and sustain nutritional ketosis. The diet effectively shifts the primary source for cellular energy production from glucose to ketone bodies. This provides an alternative fuel source for the brain and other tissues, which may be particularly beneficial to those with conditions associated with disrupted neural metabolism. Originally developed in the 1920s as a treatment for epilepsy in children, the KD demonstrated a significant effect in reducing seizure frequency and severity when pharmacological intervention had failed [1]. Over the years, its use has extended beyond epilepsy, with growing interest in its potential benefits for mental health disorders and neurodegenerative diseases [2]. The KD, through marked carbohydrate restriction, promotes stable glycemic control and minimizes acute glucose excursions. This is relevant to central nervous system (CNS) function because fluctuations in blood glucose may influence neuronal excitability and metabolic stress. By reducing reliance on glycolysis and limiting rapid increases in circulating glucose, the KD may help stabilize neuronal energy supply while decreasing downstream effects such as oxidative stress and excitatory neurotransmission.

**Figure 1:**
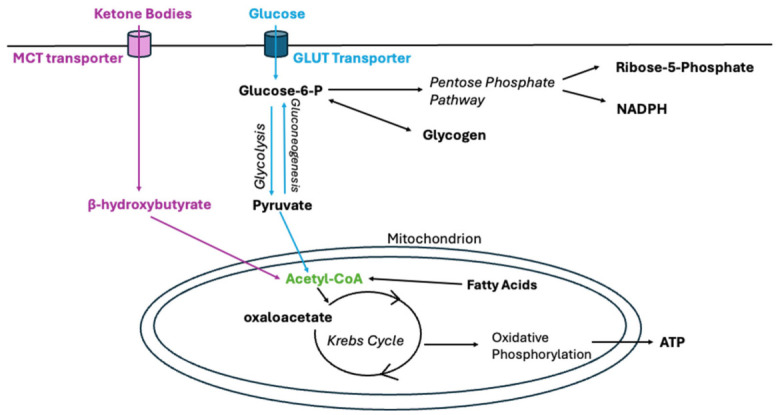
Schematic representation of the major metabolic pathways contributing to cellular energy production. Glucose enters the cell through GLUT transporters and is converted through glycolysis into pyruvate, which feeds into the mitochondrial Krebs cycle when oxaloacetate is available. Glycogen serves as an intracellular glucose reserve, while the pentose phosphate pathway generates ribose-5-phosphate and NADPH (Nicotinamide adenine dinucleotide phosphate) for biosynthesis and redox balance. Fatty acids and ketone bodies enter mitochondria and are converted to acetyl-CoA and contributing to the Krebs cycle. Ketone bodies enter a cell via a monocarboxylate transporter (MCT). All pathways converge on oxidative phosphorylation to produce ATP.

To understand the diet’s effects, it is important to first consider the body’s usual mechanism for energy production in the form of adenosine triphosphate (ATP) (Figure 1). A primary substrate is glucose, which first converts to glucose-6-phosphate, which serves as a substrate for multiple different cellular processes, including the pentose phosphate pathway and glycolysis. In glycolysis, glucose-6-phosphate is converted to pyruvate through multiple steps and then becomes acetyl-CoA, a key substrate for the Krebs cycle (also known as the TCA cycle or the citric acid cycle). The Krebs cycle produces reducing equivalents that drive the electron transport chain, leading to the process of oxidative phosphorylation that produces ATP, the body’s primary energy currency that fuels cellular activities [3].

When glucose is abundant, the body stores the excess carbons in the form of glycogen or fat for future use. Consumption of glucose or carbohydrates signals the release of insulin, a hormone released by the pancreas that activates the uptake of glucose, promoting its conversion to its storage form of glycogen, primarily in liver and skeletal muscle. Insulin also stimulates the conversion of glucose into glycerol-3-phosphate, which is essential for esterifying fatty acids into triacylglycerols (triglycerides) for fat deposition [4]. As a result of starvation, or a ketogenic diet, plasma glucose levels drop, and glucose is replenished either from glycogen breakdown or from gluconeogenesis using small molecules such as pyruvate or lactate in organs such as the liver. The pancreas reduces the level of insulin release as a response to low glucose levels while increasing the release of glucagon to activate gluconeogenesis and glycogenolysis. Fatty acids are also oxidized in response to starvation, producing acetyl-CoA and NADH (Nicotinamide adenine dinucleotide). The NADH can fuel the electron transport chain and ensure ATP production.

After depleting stored glycogen after about a day of starvation, the rate of gluconeogenesis becomes insufficient to maintain blood glucose levels, and ketogenesis is stimulated to provide ketone bodies as an alternative source of energy [5,6]. Oxaloacetate is a precursor for gluconeogenesis, so when the plasma glucose is low, oxaloacetate is drawn away from the Krebs cycle to support gluconeogenesis, especially in the liver, to resupply glucose. As a result, acetyl-CoA cannot enter the Krebs cycle, so acetyl-CoA subsequently accumulates. The high levels of acetyl-CoA lead to the production of ketone bodies, which come in three major forms: Beta-hydroxybutyrate (β-OHB), acetoacetate, and acetone. Ketone body levels can be estimated using plasma β-OHB, which is 0.05–0.1 mM at baseline and rises above 0.5 mM in ketosis [7]. Accumulation of acetyl-CoA leads directly to the formation of acetoacetate, and then β-OHB, depending on the availability of NADH. A small fraction of the acetoacetate undergoes non-enzymatically decarboxylation to form acetone, which is a volatile compound that is either exhaled in the breath or excreted in urine [8]. Ketone bodies are distributed via the bloodstream to extrahepatic organs to be reconverted to acetyl-CoA, which can be used to produce energy in cells that are not deprived of oxaloacetate.

Even though the human brain accounts for 2% of body weight, it uses about 20% of glucose-derived energy even at its resting state when there is limited neurotransmitter activity and less need for neurotransmitter synthesis [9]. About 70% of brain energy expenditures are utilized for neuronal signaling, from maintaining resting potential and firing action potential to activating postsynaptic receptors [10,11]. The brain stores only a scarce amount of glycogen, thus needing a continuous supply of glucose to maintain the brain’s cellular function [12]. When there is no adequate glucose available for the brain to utilize, whether it is during fasting or on low carbohydrate diets, including the ketogenic diet, ketone bodies (acetoacetate and β-OHB), pyruvate, and lactate can become the main alternative energy source for the brain. These fuels can cross the blood–brain barrier (BBB) via monocarboxylate transporters (MCTs) embedded within endothelial cells and astrocytes [13,14]. When fasting is prolonged for about 5–6 weeks, the ketone body levels increase significantly and replace glucose as the brain’s predominant energy source [15]. Once acetoacetate and β-OHB have crossed the BBB, in these extrahepatic tissues, they are converted back to acetyl-CoA to enter the Krebs cycle to culminate in the generation of ATP.

The ketogenic diet (KD) is a change in nutritional consumption to increase the daily intake of fat while drastically reducing the intake of carbohydrates. Thus, in some ways, the ketogenic diet mimics the starvation state. The application of KD adapts cells to use fatty acids as a source of energy rather than carbohydrates, which increases the production of ketone bodies, thus mimicking a starvation state [16]. The diet was developed over a hundred years ago for patients with epilepsy in response to the limited treatment available, particularly for children with refractory epilepsy [17]. Since then, multiple studies have confirmed the clinical application of the ketogenic diet in patients with epilepsy. For example, in a 1998 multicenter study with 51 children with drug-resistant epilepsy, 50% of those who maintained the ketogenic diet for a year reported a 90 percent decrease in seizures, 37 percent had a 50 to 90 percent decrease in seizures and five children reported being free of seizures [18]. Another study with 150 children with epilepsy found that after one year of diet, seven percent reported no seizures, and 27% had a decrease in seizure frequency of more than 90 percent [19]. In 2008, one of the first randomized clinical controlled trials was conducted to assess the efficacy of the ketogenic diet on children with epilepsy and drug-resistant to anti-epileptic drugs and at three months showed a significant 75% reduction in seizures for those in the diet group than in the controls [1]. During the last decade, interest in ketogenic treatments has expanded beyond the management of drug-refractory epilepsy. The ketogenic diet has been reported to potentially improve cardiovascular health [20], suppress cancer [21], and show clinical benefit in patients with neurological and mental disorders, the latter being the focus of this review. Unlike prior reviews emphasizing clinical breadth, this review focuses on biochemical mechanisms underlying ketogenic diet efficacy in schizophrenia, bipolar disorder, and Alzheimer’s disease [2,22,23].

## 2. Biochemical Alterations Associated with the Ketogenic Diet

Over the past several decades, research has explored the molecular mechanisms that appear to underlie the therapeutic effects of the ketogenic diet. Although its mechanism of action is not yet fully understood, current evidence suggests that its benefits arise from multiple interacting pathways rather than a single process. Studies in epilepsy models, for example, propose that the ketogenic diet may reduce activity in the mammalian target of rapamycin (mTOR) signaling pathway, which plays an important role in regulating cellular growth, metabolism, and homeostasis [24]. Findings from both laboratory and clinical research indicate that ketogenic states can influence mTOR-related signaling dynamics and may contribute to the diet’s neuroprotective properties, offering one possible explanation for its observed effectiveness across several neurological conditions [25,26].

**Figure 2:**
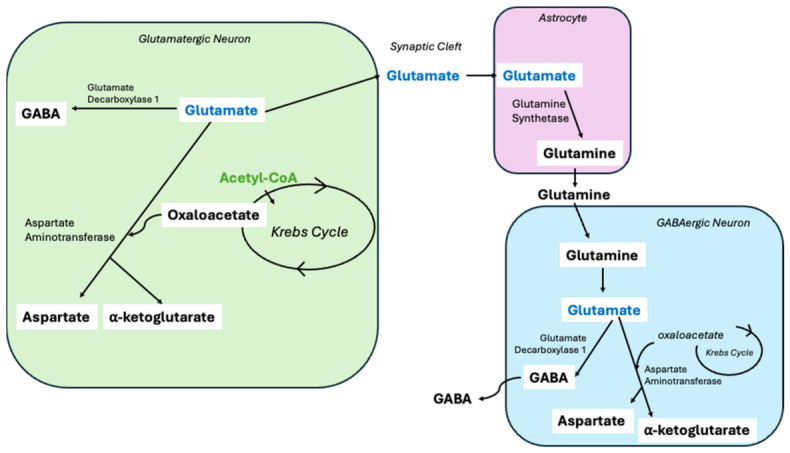
Neuron–astrocyte glutamate–glutamine cycle coupled to GABA synthesis and the Krebs cycle. Glutamatergic neurons, GABAergic neurons, and astrocytes are coupled metabolically. In glutamatergic neurons, synaptically released glutamate is taken up by astrocytes, where it is converted to glutamine by glutamine synthetase. Glutamine is exported back to neurons and reconverted to glutamate, replenishing neurotransmitter pools. In GABAergic neurons, glutamate serves as the precursor for GABA synthesis, while Krebs cycle intermediates (oxaloacetate and α-ketoglutarate) interconvert via transamination reactions linked to aspartate metabolism. Acetyl-CoA entry into the Krebs cycle supports neurotransmitter synthesis in both neuronal populations, highlighting tight metabolic integration among neurons and astrocytes during excitatory and inhibitory neurotransmission.

The ketogenic diet (KD) may influence neurotransmission in part by altering the balance between γ-aminobutyric acid (GABA) and glutamate [27]. Glutamate is an excitatory neurotransmitter and a precursor for several metabolic pathways. In the glutamate-glutamine cycle, glutamatergic neurons release glutamate into the synapse, where it is taken up by astrocytes and converted into glutamine. Astrocytes then release the glutamine back into the extracellular space, allowing absorption by neurons and regeneration back into glutamate (Figure 2). Glutamate can also be deaminated to α-ketoglutarate via glutamate dehydrogenase, decarboxylated to GABA, an inhibitory neurotransmitter, through glutamate decarboxylase 1. Additionally, it may be transaminated to aspartate through aspartate aminotransferase, a reaction that requires oxaloacetate as a nitrogen acceptor [28]. Under KD conditions, the brain shifts away from cytosolic glucose metabolism toward mitochondrial oxidation of ketone bodies and acetate. This enhances acetyl-CoA availability, increasing its condensation with oxaloacetate via citrate synthase to drive the TCA cycle. By drawing oxaloacetate into the TCA cycle, less is available for the transamination of glutamate to aspartate. As a result, more glutamate is directed toward GABA synthesis, thus lowering the glutamate/GABA ratio [29].

Another potential benefit of the ketogenic diet is the observed reduction in oxidative stress in a cell or organ. Reactive oxygen species (ROS), including superoxide and hydroxyl radicals, play a central role in cellular redox balance but can cause damage when produced in excess. Glutathione (GSH), a key antioxidant molecule, plays an essential role in maintaining cellular redox balance by neutralizing reactive oxygen species (ROS) and reactive nitrogen species (RNS) both directly and by serving as a cofactor for antioxidant and detoxification enzymes that convert ROS and RNS into non-harmful molecules, preventing cellular damage [30]. Several preclinical studies in mouse and rat models have reported increased glutathione levels in animals fed a ketogenic diet. For example, one study in adolescent male rats demonstrated elevated hippocampal glutathione, increased mitochondrial GSH, and enhanced de novo GSH synthesis. These changes were accompanied by improved hippocampal redox status, reduced mitochondrial hydrogen peroxide production, and a lower frequency of oxidative lesions, with no evidence of mitochondrial DNA damage [31,32]. Building on these preclinical findings, clinical research in humans following adherence to a ketogenic diet has also reported increased brain GSH levels [33].

Another mechanism by which KD may affect antioxidant mechanisms is through modulation of miRNA expression. In a study of obese individuals following a low-carbohydrate KD (carbohydrate intake below 30 g per day), KD altered circulating miRNA expression with several miRNAs shifting toward the levels seen in lean individuals [34]. Notably, hsa-miR-548d-3p was upregulated after KD in obese individuals. By bioinformatic analysis, hsa-miR-548d-3p is predicted to interact with the expression of superoxide dismutase 2 (SOD2). Because SOD2 is a mitochondrial antioxidant enzyme that converts superoxide radicals into hydrogen peroxide, this interaction suggests a role in oxidative stress homeostasis [35]. Some other miRNA levels changed after KD are also linked to the antioxidant pathway by enzymes such as glutathione peroxidase (GPX7), predicted to be involved in cellular response to oxidative stress. These findings are also consistent with pre-clinical studies that show changed glutathione peroxidase activity, suggesting that KD modulates antioxidant enzyme expression [36].

Under a diet that is high in carbohydrates, typically 250 g of carbs per day, glucose metabolism drives ATP production primarily through glycolysis. In contrast, the ketogenic diet allows cells to bypass much of glycolysis by relying on ketone body metabolism, thereby reducing the cells’ dependence on glucose. Importantly, ketone catabolism to acetyl-CoA generates less NADH. Reducing glycolytic flux helps lower oxidative stress, as glycolysis can produce excess NADH that feeds the electron transport chain during the reduction of molecular oxygen. In the electron transport chain, elevated NADH levels increase electron leakage at complexes I and III, leading to the premature reduction of oxygen and the formation of superoxide, a primary reactive oxygen species. Excess ROS, produced as a byproduct of electron transport, can lead to redox imbalance in the cell. While the metabolism of one glucose molecule reduces two molecules of NAD^+^ to NADH during glycolysis and two more during the pyruvate dehydrogenase reaction, the oxidation of β-OHB to acetyl-CoA reduces only one molecule of NAD^+^ through β-OHB dehydrogenase, placing a lower demand on NAD^+^ to generate equivalent metabolic precursors. By shifting the brain’s primary fuel source from glucose to ketones, the cellular requirement for NAD^+^ reduction diminishes, thereby preserving NAD^+^ and substantially increasing the NAD^+^/NADH ratio. This higher ratio promotes the activity of NAD^+^-dependent enzymes and enhances the thermodynamic efficiency of the mitochondrial electron transport chain, ultimately limiting superoxide formation and supporting redox homeostasis [37].

**Figure 3:**
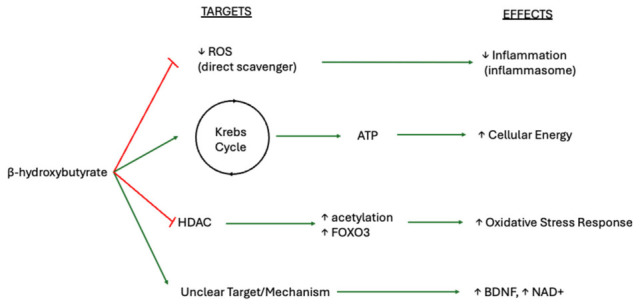
Proposed cellular targets and downstream effects of β-hydroxybutyrate (β-OHB). The schematic illustrates key molecular targets and signaling pathways influenced by β-hydroxybutyrate. β-OHB directly scavenges reactive oxygen species (ROS), leading to reduced inflammasome activation and decreased inflammation. As a metabolic substrate, β-OHB enters the Krebs cycle, increasing ATP production and cellular energy availability. β-OHB also inhibits histone deacetylases (HDACs), resulting in increased protein acetylation and activation of the FOXO3 pathway, which enhances oxidative stress responses. Additional effects mediated through currently unclear targets or mechanisms include increased brain-derived neurotrophic factor (BDNF) and NAD^+^ levels. Green arrows indicate stimulatory effects, while red inhibitory symbols indicate suppression of targets.

β-Hydroxybutyrate (β-OHB) and acetoacetate, two of the primary ketone bodies, may also reduce oxidative stress by directly scavenging reactive species (Figure 3). Both have been reported to neutralize hydroxyl radicals, while acetoacetate can additionally scavenge hypochlorous acid, singlet oxygen, and—importantly—peroxynitrite, a highly reactive oxidant derived from nitric oxide [38,39]. Beyond direct radical scavenging, β-OHB functions as an inhibitor of class I histone deacetylases (HDACs). In preclinical studies, HDAC inhibition by β-OHB increased transcriptional activity of genes involved in oxidative stress resistance, including FOXO3A and MT2, accompanied by enhanced histone acetylation at their promoters due to reduced HDAC1 activity. The same study also evaluated lipid peroxidation, a downstream consequence of oxidative stress, and found markedly elevated levels of 4-hydroxynonenal. This increase was significantly suppressed in mice pretreated with β-OHB, indicating that ketone bodies confer protection against both oxidative stress and lipid peroxidation [7,40].

Beyond indirect antioxidant signaling, ketones—particularly β-hydroxybutyrate—also exert a direct suppressive effect on the generation of reactive oxygen species at the level of mitochondrial complexes. In addition to reducing ROS generation at mitochondrial complex I, β-hydroxybutyrate enhances the expression of uncoupling proteins which help optimize electron transport chain flux during oxidative phosphorylation. By mildly dissipating the mitochondrial membrane potential, these uncoupling proteins allow protons to re-enter the mitochondrial matrix more readily, decreasing electron leak and lowering the probability that electrons will prematurely react with molecular oxygen to form superoxide radicals. Through this mechanism, β-OHB contributes to more stable mitochondrial redox conditions and lowers oxidative stress [41].

HDAC inhibition by β-hydroxybutyrate is associated with an increased expression of neuroprotective genes, including brain-derived neurotrophic factor. Elevated BDNF levels can enhance neuronal plasticity but may also influence neuroimmune interactions, as BDNF signaling can activate microglia and astrocytes—two major cell types involved in neuroinflammatory pathways, particularly in cognitive brain regions such as the hippocampus and cortex [42,43]. However, BDNF regulation is not uniform across brain regions. In mice, β-OHB administration increases BDNF expression in the hippocampus, and both BDNF protein and mRNA levels are elevated in cultured hippocampal neurons. These findings suggest that β-OHB promotes BDNF-related signaling in hippocampal models via the cAMP/PKA/p-CREB pathway [44]. Fasting-related metabolic states similarly modulate BDNF-linked signaling and autophagy in a region-specific manner, affecting the cortex, hippocampus, and hypothalamus differently. Elevated BDNF may further influence neuroimmune interactions by activating microglia and astrocytes involved in neuroinflammatory pathways [45]. Thus, while β-OHB-mediated HDAC inhibition supports antioxidant and metabolic resilience, its role in BDNF-linked glial activation highlights a complex regulatory network through which ketone bodies may shape neuroinflammatory responses in a region or context-dependent manner.

## 3. Metabolic Dysfunction in Schizophrenia: Implications for Ketogenic Therapy

Among the neurological disorders that may benefit from the therapeutic effects of the ketogenic diet are schizophrenia spectrum disorders. Schizophrenia is a complex neuropsychiatric condition characterized by disturbances in perception, sensory experiences (such as hallucinations), and cognition (including delusions) [46]. Symptoms can be divided into two categories: negative and positive. Negative psychotic symptoms involve a reduction or absence of normal behaviors and emotional responses, such as avolition (a lack of motivation to engage in goal-oriented activities) and apathy. In contrast, positive symptoms consist of behaviors or perceptions that are not normally present in healthy individuals, including hallucinations, delusions, and paranoia.

### 3.1. Molecular Genetic Risk Factors and Neurotransmitter Dysregulation

The exact causes of schizophrenia remain unknown; however, strong evidence points to genetic risk factors that contribute to disease susceptibility. One of the most well-established molecular genetic risk factors for schizophrenia is chromosome 22q11.2 deletion syndrome (22q11DS). The deleted region on chromosome 22 contains several critical genes, including COMT and PRODH [47]. The COMT gene encodes catechol-O-methyltransferase, an enzyme responsible for the catabolism of dopamine, a neurotransmitter closely associated with mood regulation, motor control, and cognitive function. Reduced COMT activity can lead to elevated dopamine levels, and dysregulation of dopamine signaling is considered central to the pathophysiology of schizophrenia. Consequently, abnormal COMT function may influence both cognitive performance and vulnerability to environmental stressors. In addition to deletion-related COMT deficiency, the COMT Val158Met polymorphism has also been studied in individuals with schizophrenia and is associated with altered enzyme activity, further contributing to cognitive dysfunction through its effects on dopamine regulation in the prefrontal cortex [48]. The PRODH gene encodes proline dehydrogenase, an enzyme involved in proline metabolism, and its deficiency has likewise been linked to cognitive impairment. Unlike COMT, PRODH influences neurotransmission by regulating glutamatergic signaling through the conversion of proline to glutamate. Glutamate is the primary excitatory neurotransmitter in the central nervous system and plays a critical role in learning, neurodevelopment, and the balance of other neurotransmitter systems, including GABA and dopamine. Reduced PRODH activity may lead to proline accumulation, which can disrupt normal glutamate signaling. Aberrant glutamatergic neurotransmission has been strongly associated with schizophrenia. Together, alterations in glutamatergic signaling due to PRODH deletion and dopaminergic dysregulation resulting from COMT disruption within the 22q11.2 region may represent key contributors to the pathophysiology observed in 22q11DS-associated schizophrenia [47].

The genetic contributions to schizophrenia extend well beyond 22q11.2 deletion syndrome, with more than 130 genomic loci reported to be associated with a significantly increased risk of developing the disorder [49]. Many of these genes do not encode proteins that directly regulate gene expression; instead, they influence synaptic function, neuronal connectivity, and neurotransmitter signaling. Notably, approximately 30% of identified schizophrenia-associated genes are involved in glutamatergic synapses, particularly those related to N-methyl-D-aspartate (NMDA) receptors, which are essential for synaptic plasticity and cognitive processes such as learning and memory formation [49]. Dysfunctions in NMDA receptor signaling have been widely implicated in the pathophysiology of schizophrenia, arising from both genetic alterations and environmental stressors that impair receptor function and cognitive processing. In addition to glutamatergic abnormalities, disruptions in dopamine signaling are a well-established feature of the disorder. Studies have shown that hyperactivity in the mesolimbic dopamine pathway and reduced dopamine activity in the mesocortical pathway both contribute to distinct symptom profiles of schizophrenia [50]. Together, these findings underscore schizophrenia as a disorder arising from complex interactions between multiple neurotransmitter systems—particularly glutamate and dopamine—whose regulation can be influenced by diverse genetic variations, including COMT and PRODH dysregulation as observed in 22q11DS.

### 3.2. Oxidative Stress and Bioenergetic Impairment

In schizophrenia, elevated ROS generation coupled with impaired antioxidant defenses is thought to contribute to mitochondrial dysfunction, disruption of neurotransmitter pathways, and abnormal energy metabolism. This oxidative stress damages lipids, proteins, and DNA, leading to cellular dysfunction and tissue injury. The systemic nature of oxidative stress in schizophrenia is of particular concern, as it may exacerbate common comorbidities such as cardiovascular disease and metabolic disturbances, including hyperglycemia resulting from impaired glucose tolerance or insulin resistance. Supporting this systemic involvement, in individuals, elevated ROS levels have been detected in peripheral tissues and urine [51], while levels of glutathione are reduced in plasma [52]. Glutathione, one of the body’s primary antioxidants, maintains cellular redox balance by directly neutralizing ROS and by serving as a cofactor for antioxidant enzymes that convert ROS into non-toxic molecules. Consequently, reduced glutathione levels reflect a compromised antioxidant defense system that increases vulnerability to ROS-mediated cellular damage [52].

Oxidative stress in schizophrenia also affects the central nervous system, particularly neurotransmitter systems critical to cognitive and behavioral regulation. Dopamine and glutamate signaling—both central to schizophrenia pathophysiology—are highly sensitive to oxidative imbalance. Dopamine metabolism through enzymatic degradation generates hydrogen peroxide, and dopamine auto-oxidation further contributes to ROS formation [38]. When antioxidant capacity is overwhelmed, ROS accumulation can deplete protective molecules such as superoxide dismutase and glutathione. Notably, glutathione enhances GABA-mediated inhibitory signaling; thus, reduced glutathione availability can impair GABAergic function, weakening inhibitory feedback in the prefrontal cortex and potentially contributing to positive symptoms of schizophrenia [53]. Oxidative stress has also been shown to influence glutamatergic signaling, particularly NMDA receptor function. According to the glutamate hypothesis of schizophrenia, impaired NMDA receptor activity reduces calcium influx, triggering compensatory increases in intracellular calcium via non-NMDA glutamate receptors and certain G-protein-coupled receptors. This dysregulated calcium signaling can further stimulate reactive oxygen species production, establishing a self-reinforcing cycle of oxidative damage [54]. Excessive ROS generation ultimately disrupts neuronal integrity, impairs synaptic plasticity, and compromises cognitive function.

In addition to neurotransmitter dysregulation, impaired energy metabolism is increasingly recognized as a key component of schizophrenia pathophysiology. As neurons are particularly dependent on efficient energy production to sustain their activity, mitochondrial dysfunction can significantly impair neuronal function. Growing evidence in schizophrenia indicates impaired mitochondrial activity, leading to reduced ATP production and compromised neuronal performance. Notably, postmortem studies have reported reduced mitochondrial size in oligodendrocytes, which may disrupt myelin formation in the frontal cortex and contribute to cognitive and executive dysfunction observed in schizophrenia [55,56].

Oxidative phosphorylation occurs within the mitochondrial electron transport chain, which consists of multiple protein complexes, including Complex I, a critical component of cellular respiration. One proposed mechanism underlying mitochondrial dysfunction in schizophrenia is reduced activity of NADH dehydrogenase flavoprotein 2 (NDUFV2), a subunit of Complex I. Dysfunction of NDUFV2 impairs Complex I activity, resulting in decreased mitochondrial energy production. Reduced NDUFV2 activity has been observed in individuals with schizophrenia, potentially due to interference by elevated expression of NDUFV2P1, a pseudogene of NDUFV2 [57]. Importantly, the NDUFV2 gene is located on chromosome 22, and its dysfunction may also be linked to chromosome 22q11.2 deletion syndrome, further supporting a genetic contribution to mitochondrial impairment in schizophrenia [58].

Furthermore, impaired glycolytic metabolism in the brain has also been implicated in schizophrenia. Recent studies have reported decreased activity of key glycolytic enzymes—particularly hexokinase and phosphofructokinase—in the dorsolateral prefrontal cortex of individuals with schizophrenia [59]. These enzymes play critical roles during the investment phase of glycolysis: hexokinase catalyzes the phosphorylation of glucose to glucose-6-phosphate, the first step of the pathway, while phosphofructokinase regulates the rate-limiting step of glycolysis. Reduced activity of these enzymes suggests dysfunction within the glycolytic pathway, which would impair the brain’s ability to efficiently generate ATP—an especially critical issue given the brain’s high reliance on glucose as its primary energy source. When glycolytic dysfunction occurs alongside mitochondrial impairment, the resulting energy deficit can significantly compromise neural function.

In addition to limiting energy production, combined mitochondrial and glycolytic dysfunction may further exacerbate oxidative stress. Reduced ATP availability may force compensatory increases in metabolic activity, placing additional strain on already compromised mitochondria. Under these conditions, irregular electron flow through the electron transport chain can increase the generation of reactive oxygen species. Moreover, glycolysis supplies intermediates for other metabolic pathways, including the pentose phosphate pathway, which produces nicotinamide adenine dinucleotide phosphate (NADPH), an essential cofactor for antioxidant defenses. NADPH is required for the regeneration of antioxidants such as glutathione, which plays a central role in neutralizing ROS. Disruption of glycolysis can therefore reduce NADPH availability, weaken antioxidant capacity, and further destabilize cellular redox balance. Collectively, these effects can increase oxidative stress, leading to neuronal and glial damage, impaired synaptic function, reduced neuroplasticity, and ultimately neurodegeneration [59].

### 3.3. Pharmacological Treatments and the Rationale for Ketogenic Therapy

Treatment options for schizophrenia have historically focused on symptomatic relief. The first antipsychotic drug, chlorpromazine, is a typical antipsychotic that works primarily by antagonizing dopamine-2 (D2) receptors. This prototypical drug was effective in managing some positive symptoms, such as hallucinations, delusions, and anxiety, but was often less effective in treating negative symptoms, including social withdrawal and cognitive deficits [60]. Moreover, D2 receptor antagonist antipsychotics tend to have limited selectivity. As a result, in addition to blocking receptors at the intended target sites, they also antagonize dopamine receptors in other dopaminergic pathways. For example, blockade of D2 receptors in the nigrostriatal pathway can lead to extrapyramidal side effects, such as tremors and bradykinesia. Antipsychotic treatment may also reduce patients’ ability to experience pleasure by blocking D1 receptors in the mesolimbic reward pathway, potentially exacerbating negative symptoms and contributing to anhedonia (the inability to experience pleasure) or emotional blunting [60,61].

The newest generation of antipsychotic drugs, often referred to as third-generation antipsychotics, act as partial agonists at D2 receptors rather than as full antagonists. When extracellular dopamine levels are high, these partial agonists compete with dopamine and produce a net antagonistic effect. Conversely, when dopamine levels are low, they bind to additional receptors and partially activate them. This mechanism is thought to provide improved symptom management by modulating, rather than completely blocking, dopamine receptor activity. Although third-generation antipsychotics are associated with fewer extrapyramidal symptoms and lower rates of akathisia (a sensation of inner restlessness accompanied by an urge to move), dystonia, and tardive dyskinesia (a drug-induced movement disorder characterized by involuntary facial and bodily movements, typically after long-term antipsychotic use) compared with first-generation antipsychotics, side effects are not fully eliminated. These medications can still cause sedation, akathisia, weight gain, hyperprolactinemia, QTc prolongation, nausea, constipation, and dizziness [62,63,64]. Such adverse effects frequently necessitate additional pharmacological treatment or, if severe, lead patients to discontinue therapy, increasing the risk of relapse. Furthermore, antipsychotic medications are often prescribed using a trial-and-error approach, which can take weeks to months to identify an effective dosage that balances symptom control with tolerable side effects. Despite treatment, approximately 20–67% of patients with schizophrenia show little or only partial response to antipsychotic medications [65].

Given the side effects and limitations of current antipsychotic treatments, researchers are increasingly exploring alternative therapeutic approaches for schizophrenia. One option that has gained growing interest is the ketogenic diet, with several case studies and pilot trials reporting reductions in schizophrenia symptoms. Notably, in a four-month pilot study, participants with schizophrenia who followed a ketogenic diet of 10% carbohydrate, 30% protein, and 60% fat demonstrated a 32% reduction in scores on the Brief Psychiatric Rating Scale [66]. Although the precise mechanisms underlying the ketogenic diet’s effects remain unclear, several hypotheses have been proposed. As previously discussed, schizophrenia is characterized by a deficit in glutathione, which contributes to reactive oxygen species accumulation and reduced GABAergic activity. Włodarczyk et al. hypothesize that the ketogenic diet—defined as low-carbohydrate (about 30–50 g per day), medium-protein (up to 1 g/kg daily) and high-fat intake (around 80% of daily calories)—may help counteract these deficits by increasing the GABA-to-glutamate ratio [67]. As discussed in Section 2 and illustrated in Figure 2, ketogenic metabolism shifts glutamate utilization toward GABA synthesis [29]. Elevated GABA levels may therefore help compensate for reduced GABAergic activity associated with glutathione deficiency.

Patients with schizophrenia also exhibit disruptions in energy metabolism due to mitochondrial dysfunction and impaired glycolytic pathways. By bypassing dysfunctional glycolytic steps (Section 2), the ketogenic diet may mitigate neuronal energy deficits in schizophrenia. In addition, the ketogenic diet has been shown to increase mitochondrial glutathione levels and stimulate de novo glutathione (GSH) biosynthesis. In one study using adolescent male rats, a ketogenic diet (Bio-Serv F3666—6.3:1 ratio of fat:carbohydrate plus protein) led to increased glutathione levels in the hippocampus [31]. This increase was accompanied by improved redox status, reduced hydrogen peroxide production in hippocampal mitochondria, and lower frequencies of oxidative lesions, indicating reduced mitochondrial DNA damage [31].

Beyond its role in redox regulation, increased glutathione may also help restore diminished GABAergic activity, as glutathione has been shown to induce GABA release [68]. As previously discussed, NMDA receptor dysfunction represents another key pathophysiological feature of schizophrenia. In an animal model of schizophrenia characterized by NMDA receptor hypofunction, administration of a ketogenic diet rescued deficits in sensorimotor gating. Sensorimotor gating is a behavioral process involved in filtering irrelevant stimuli, and impairments in this process are closely associated with psychosis. In this model, MK-801—a potent NMDA receptor antagonist—was used to induce prepulse inhibition deficits, a widely used behavioral measure of sensorimotor gating in both animals and humans. By blocking NMDA receptors, MK-801 mimics glutamatergic hypofunction, which is believed to contribute to cognitive impairments observed in schizophrenia. The observed reversal of prepulse inhibition deficits suggests that the ketogenic diet can ameliorate a core schizophrenia-related impairment by improving sensorimotor gating [69].

Although many of the studies discussed above are based on animal models that may not fully translate to human conditions, the potential benefits of the ketogenic diet for patients with schizophrenia warrant consideration. Improvements observed in preclinical models—including enhanced redox balance, improved mitochondrial function, and better regulation of neurotransmitter systems—suggest that this dietary approach may address several core pathophysiological features of schizophrenia, such as oxidative stress, energy deficits, and neurotransmitter dysregulation.

Moreover, the ketogenic diet in the classic formulation or the medium-chain triglyceride version has been shown to be safe for long-term use in humans, particularly in the treatment of epilepsy [70]. Unlike many antipsychotic medications, which are often associated with weight gain and metabolic disturbances, the ketogenic diet does not carry these same risks. However, it is important to note that the diet may lead to weight loss in some individuals, which could be detrimental for patients who are already underweight. While the KD shows favorable effects on glucose metabolism and, in some cases, lipid profiles, we note that long-term adherence may be associated with elevations in low density lipoprotein (LDL) cholesterol in certain individuals, requiring careful cardiovascular risk assessment and monitoring. There are potential hepatic considerations, including increased fat handling and the risk of steatosis in susceptible individuals, as well as the need to monitor liver function during extended use. Practical considerations include gastrointestinal side effects and the importance of appropriate dietary formulation to mitigate these risks. Due to its restrictions, particularly on fruit, supplementation with vitamins and other micronutrients may be necessary [6]. Many of these adverse effects may be context-dependent and can be minimized through careful patient selection, medical supervision, and individualized diet design. While the KD has an established safety record in neurological populations such as epilepsy, its long-term safety profile in psychiatric and neurodegenerative populations remains less well defined and warrants further controlled study.

While the ketogenic diet in its various formulations, including the modified Atkins-style diets and supplementation with exogenous ketones, is unlikely to serve as a standalone treatment for schizophrenia, early clinical data provide a strong rationale for further investigation [71]. When used adjunctively with conventional therapies, the ketogenic diet may offer additional therapeutic benefits. Nevertheless, adherence to the diet can be challenging due to its strict dietary requirements and the need for careful planning and support. Despite these challenges, the potential therapeutic value of the ketogenic diet, combined with encouraging preclinical findings and emerging case studies, underscores the need for continued exploration.

## 4. Metabolic Dysfunction in Bipolar Disorder: Implications for Ketogenic Therapy

Bipolar disorder (BD) affects an estimated 40 million people worldwide, with prevalence increasing in recent decades [72]. It is a psychiatric and neurological disorder characterized by alternating episodes of mania or hypomania—marked by elevated mood, increased energy, and activity—and depression, involving low mood, reduced energy, and loss of interest. Bipolar disorder is classified into several subtypes, including bipolar I disorder, bipolar II disorder, and cyclothymic disorder. Bipolar I disorder is defined by one or more manic episodes that may alternate with major depressive episodes, whereas bipolar II disorder involves at least one hypomanic episode alternating with major depressive episodes. Cyclothymic disorder is characterized by chronic mood instability, with hypomanic and depressive symptoms that do not meet the criteria for major mood episodes but persist for at least two years. The onset of bipolar disorder most commonly occurs during two age ranges: 15–24 years and 45–54 years. Notably, more than 70% of individuals diagnosed with bipolar disorder experience the onset of symptoms before the age of 25 [73].

### 4.1. Molecular Genetic Risk Factors and Neurotransmitter Dysregulation

Similarly to schizophrenia, the precise molecular mechanisms underlying bipolar disorder remain unclear. Current evidence suggests that BD arises from a combination of genetic, epigenetic, neurochemical, and environmental factors. Genetic studies have identified multiple chromosomal regions and candidate genes associated with increased risk for BD. Notably, genome-wide association studies (GWAS) have been widely used to identify genetic variants linked to the disorder.

Baum et al. identified a single nucleotide polymorphism (SNP) in the diacylglycerol kinase eta (η) (DGKH) gene that may be associated with BD [74]. DGKH encodes an enzyme that catalyzes the conversion of diacylglycerol (DAG) to phosphatidic acid. Reduced activity of DGKH impairs DAG catabolism, resulting in elevated intracellular DAG. DAG plays a critical role in intracellular signaling as a cofactor for multiple isoforms of protein kinase C (PKC). PKC is involved in a wide range of signal transduction pathways, including those regulating cell growth, differentiation, and synaptic signaling. Dysregulation of PKC activity has been implicated in the pathophysiology of bipolar disorder, particularly during manic episodes. Studies have reported increased PKC activity in patients with BD during manic states [75,76]. PKC also modulates several key neurochemical pathways, including the regulation of neurotransmitters such as dopamine and serotonin. One study observed increased PKC translocation in association with serotonin signaling in individuals with BD [77]. Together, these findings suggest that DGKH-related signaling pathways contribute to bipolar disorder, especially in manic phases.

In addition to DGKH, other GWAS have identified associations between BD and the CACNA1C gene, which encodes subunits of voltage-gated calcium channels, suggesting that altered calcium signaling may contribute to BD pathophysiology [78]. Associations have also been reported for the ANK3 gene, which plays a role in stabilizing and clustering ion channels at the nodes of Ranvier. These findings support the hypothesis that disruptions in ion channel function and neuronal excitability are components of the molecular mechanisms underlying bipolar disorder [79,80].

Another pathological feature of bipolar disorder involves changes in neurotrophin levels compared to healthy controls. Notably, there are decreased levels of the brain-derived neurotrophic factor (BDNF) observed in patients with bipolar disorder [81,82]. Reduced BDNF levels are associated with several key aspects of BD, including neuroprogression, impaired neuroplasticity, reduced stress resilience, and increased vulnerability to stress. BDNF plays a critical role in regulating synaptic plasticity, which is essential for normal neuronal function and adaptability. It promotes the phosphorylation of synapsin, a protein involved in synaptic vesicle release, thereby increasing the release of neurotransmitters such as glutamate and GABA [83]. This process enhances synaptic communication between neurons. Additionally, BDNF strengthens synaptic connections by facilitating ion influx through NMDA receptors, which, as discussed previously, are central to synaptic plasticity and synaptic strengthening [84]. Together, these BDNF-mediated processes support mood regulation and overall brain function. Consequently, alterations in BDNF expression or secretion can impair synaptic plasticity, disrupt neurotransmitter release and stability—particularly in mood-regulating circuits—and contribute to the development and persistence of bipolar disorder symptoms.

Alterations in neurotransmitter pathways also play a central role in the pathophysiology of bipolar disorder. Multiple lines of evidence—including animal models, postmortem analyses, in vivo imaging, and functional MRI (fMRI) studies—have demonstrated changes in dopamine levels and dopaminergic receptor expression. Notably, in vivo imaging studies of patients experiencing mania have shown increased dopamine D2 receptor availability [85]. Alterations in GABA signaling have also been reported in BD. Elevated GABA levels have been observed in euthymic patients with bipolar disorder [86], whereas reduced GABA concentrations have been detected in the plasma and cerebrospinal fluid of patients with BD [87,88]. Postmortem studies further reveal abnormalities in glutamatergic function, affecting both presynaptic and postsynaptic signaling, as well as alterations in excitatory synaptic connectivity [89,90].

In addition, elevated glutamate levels in BD have been correlated with increased pyruvate carboxylase activity. In astrocytes, pyruvate carboxylase catalyzes the conversion of pyruvate and bicarbonate into oxaloacetate, a key intermediate of the Krebs cycle. Oxaloacetate subsequently contributes to the production of α-ketoglutarate, a precursor for *de novo* glutamate synthesis in the brain [91]. Increased pyruvate carboxylase activity in BD may therefore reflect a metabolic shift that enhances glutamate production, potentially leading to excitotoxicity and impaired neural communication.

### 4.2. Oxidative Stress and Bioenergetic Impairment

Similarly to schizophrenia, patients with BD exhibit mitochondrial dysfunction. Magnetic resonance imaging (MRI) studies support this observation by revealing abnormal mitochondrial size and impaired function in regions such as the frontal and prefrontal cortex, as well as marginal brain areas [92]. Postmortem studies further corroborate these findings, demonstrating mitochondrial morphological abnormalities in frontal and prefrontal cortical tissues from individuals with BD [93]. The metabolic consequences of mitochondrial dysfunction extend beyond structural abnormalities and include altered biochemical markers. Several studies have reported elevated lactate and glucose levels in cerebrospinal fluid, suggesting disruption of oxidative phosphorylation. Lactate accumulation, in particular, indicates insufficient ATP production through mitochondrial respiration, leading cells to rely more heavily on anaerobic glycolysis for energy generation [94,95]. Additional evidence of impaired energy metabolism includes reduced levels of phosphocreatine, which suggest prolonged stress on cellular energy reserves due to deficient ATP production [96]. Consistent with this observation, decreased mRNA expression of creatine kinase has been documented in the hippocampus and prefrontal cortex of individuals with BD. This reduction implies dysfunction of the creatine kinase–mediated phosphagen system, which plays a critical role in maintaining cellular ATP homeostasis [97]. NDUFS7—also known as the NADH–ubiquinone oxidoreductase 20 kDa subunit—is another component of complex I and plays a key role in the mitochondrial respiratory chain [98]. Reduced protein levels of NDUFS7 resulting in impaired activity of mitochondrial complex I have been observed in the prefrontal cortex of patients with BD.

Another prominent hypothesis linking bipolar disorder to mitochondrial dysfunction involves reduced activity of the sodium–potassium adenosine triphosphate pump (Na^+^/K^+^-ATPase), which is proposed to increase neuronal resting membrane potential and promote a hyperexcitable neuronal state. Increased neuronal excitability can impair the normal clearance of intracellular calcium ions, resulting in prolonged neurotransmitter release. This mechanism may help explain the elevated neuronal calcium levels observed during manic episodes of BD. In contrast, more substantial reductions in Na^+^/K^+^-ATPase activity would further elevate the resting membrane potential while simultaneously reducing action potential amplitude. This attenuation of action potential strength could limit neurotransmitter release, potentially contributing to depressive symptoms or low-energy states associated with bipolar disorder [99]. Recent studies demonstrate that mitochondrial dysfunction and associated oxidative stress directly inhibit Na^+^/K^+^-ATPase through ATP depletion and oxidative modification of pump subunits, leading to disrupted ion homeostasis and neuronal hyperexcitability, thus providing a mechanistic framework linking mitochondrial energy failure to Na^+^/K^+^-ATPase dysregulation [100].

In addition to mitochondrial dysfunction, which can increase the production of reactive oxygen species and reactive nitrogen species, the subsequent elevated DNA/RNA damage and lipid peroxidation play a significant role in the disorder’s pathophysiology [101]. Peroxynitrite can directly induce DNA and RNA strand breaks, impairing protein synthesis and disrupting normal cellular function. Furthermore, it can interfere with DNA repair mechanisms, thereby exacerbating cellular dysfunction and contributing to neurodegeneration [102]. Nitric oxide also plays a role in lipid peroxidation, a process in which free radicals attack membrane lipids, particularly polyunsaturated fatty acids. This process generates toxic byproducts such as malondialdehyde, which can damage DNA and have been linked to pathological outcomes, including neurodegeneration and cancer [103]. Moreover, peroxynitrite can further amplify lipid peroxidation by generating hydroxyl radicals, which are highly reactive and capable of attacking membrane lipids and other cellular components [104].

### 4.3. Pharmacological Treatments and the Rationale for Ketogenic Therapy

Current pharmacological management of bipolar disorder remains largely symptom focused. Management of manic episodes primarily involves three classes of medications: lithium, mood-stabilizing anticonvulsants, and antipsychotic drugs. Most second-generation antipsychotics have been approved by the FDA for the treatment of acute mania, while lithium—a long-established therapy—continues to be widely used for mania and serves as a first-line treatment due to its well-documented prophylactic effects [105].

For depressive episodes, lithium and other mood-stabilizing medications are also commonly included in treatment regimens, although additional pharmacological approaches may be considered depending on symptom severity and patient response. Medication selection and dosing often rely on a trial-and-error approach, which can delay effective symptom control and, in some cases, exacerbate mood instability.

Moreover, these treatments are associated with significant side effects. For example, the FDA has issued a black box warning for antipsychotic medications due to an increased risk of cerebrovascular adverse events, particularly in elderly patients [106]. Certain anticonvulsants, such as lamotrigine and topiramate, have also been shown to be ineffective in treating manic episodes [107]. Additionally, recent clinical trials have demonstrated limited efficacy of some antidepressants, including paroxetine [108] and bupropion [109], in bipolar disorder. A major clinical concern—especially during acute depressive phases—is the elevated risk of suicide, which often necessitates hospitalization for close monitoring and patient safety. This risk underscores the importance of effective phase-specific management and early intervention strategies [105]. Given these limitations, adjunctive approaches to traditional pharmacotherapy are being explored. One such emerging strategy is the ketogenic diet. Recent studies, including clinical pilot trials, have reported improvements in both mental health outcomes and metabolic parameters in patients with bipolar disorder following ketogenic dietary interventions [110].

Notably, preclinical studies in models of traumatic brain injury have shown that the ketogenic diet (Bio-Serv F3666; 78.8% fat, 8.4% protein, 0.8% carbohydrate, 5% fiber) enhances antioxidant capacity and provides alternative metabolic substrates that support cellular energy metabolism. These findings are particularly relevant because such models involve inhibition of mitochondrial complex I—a deficit that has also been observed in the brains of patients with bipolar disorder. Following ketogenic diet administration, improved activity of mitochondrial complexes II and III has been reported, suggesting that ketone bodies can serve as alternative energy substrates and, through antioxidant mechanisms described above, protect against oxidative stress-mediated mitochondrial dysfunction [111]. In addition to improving mitochondrial function, the ketogenic diet offers an alternative pathway for neuronal energy production. Dysregulated Na^+^/K^+^-ATPase activity has been implicated in both manic and depressive episodes of bipolar disorder and is closely tied to cellular energy availability. By enabling neurons to rely on ketone bodies for energy generation and bypass glycolysis, the ketogenic diet may help prevent energy deficits that contribute to neuronal instability and mood dysregulation.

In addition, mitochondrial dysfunction and elevated nitric oxide (NO) levels have been suggested to increase oxidative stress in bipolar disorder [39], a process that may be mitigated by the ketogenic diet. These antioxidant effects of ketone metabolism (Section 2) may be particularly relevant. The inhibition of HDAC by β-OHB also promotes the transcription of oxidative stress–resistance genes such as FOXO3A and MT2, as reported in preclinical models [40]. Although these effects were primarily observed in renal tissue, the ketogenic diet affects the brain, HDACs are expressed in neural tissue, and β-OHB readily crosses the blood–brain barrier, suggesting potential relevance for patients with BD. Supporting this hypothesis, multiple studies have shown that increased FOXO3A expression is beneficial in neurological disorders, including Alzheimer’s disease [112] and stroke [113], further implicating oxidative stress–resistance pathways as therapeutic targets relevant to bipolar disorder.

APOE4, the strongest genetic risk factor for late-onset Alzheimer’s disease, is associated with distinct metabolic phenotypes, including reduced cerebral glucose utilization and altered lipid transport [114]. Evidence indicates that APOE4 carriers may exhibit impaired cerebral glucose metabolism at earlier stages than non-carriers, reinforcing the potential relevance of metabolic interventions such as the ketogenic diet. At the same time, emerging data suggest that the APOE4 genotype may influence lipid handling and cardiovascular risk, raising the possibility that responses to high-fat diets, including ketogenic regimens, may differ between carriers and non-carriers [115]. While ketone utilization appears is preserved in APOE4 carriers—supporting the rationale for ketogenic interventions—some studies report less favorable lipid responses or increased sensitivity to dietary fat composition in this group. Thus, the APOE genotype may be an important modifier of both efficacy and safety, underscoring the need for personalized approaches and careful metabolic monitoring in APOE4-positive individuals.

Reduced levels of brain-derived neurotrophic factor (BDNF) have been consistently observed in the brains of patients with bipolar disorder, contributing to impaired synaptic plasticity and neural connectivity. Emerging evidence suggests that the ketogenic diet may counteract this deficit by increasing BDNF expression in the brain. Preclinical studies have demonstrated that ketone bodies can stimulate BDNF production [44], findings that are supported by studies in humans receiving ketogenic medium-chain triglyceride supplementation showing elevated serum proBDNF levels [116]. Although there has been debate regarding whether BDNF can cross the blood–brain barrier, recent work has identified a correlation between serum BDNF levels and hippocampal proBDNF (the precursor of BDNF), suggesting that peripheral BDNF measurements may reflect central nervous system activity [117]. Mechanistically, β-OHB has been shown to enhance BDNF transcription by activating BDNF promoters through inhibition of histone deacetylases HDAC2 and HDAC3 in the brain, linking metabolic and epigenetic regulation to neurotrophic signaling [117]. Beyond its effects on synaptic plasticity, the high-fat, moderate-protein, low-carbohydrate ketogenic diet (<50 g carbohydrate per day), has also been associated with improvements in large-scale neural network organization. Functional MRI analyses indicate that ketogenic dietary interventions exert a stabilizing effect on neural network functional connectivity, suggesting enhanced coordination and resilience of brain networks [118]. Together, these findings support the ketogenic diet as a potential modulator of neuroplasticity and network function through BDNF-dependent and epigenetic mechanisms relevant to bipolar disorder [119].

As discussed in the previous section on schizophrenia, this dietary approach enhances GABAergic activity by replenishing oxaloacetate through increased Krebs cycle activity and shifting glutamate metabolism toward GABA synthesis [120]. By reducing glutamate levels and increasing GABA availability, the ketogenic diet may help restore the balance between excitatory and inhibitory neurotransmission. This rebalancing may contribute to mood stabilization and potentially reduce the frequency or severity of both manic and depressive episodes in bipolar disorder. In summary, the ketogenic diet shows potential therapeutic benefits by improving mitochondrial function, thereby reducing oxidative stress while also enhancing synaptic plasticity and neural connectivity. As a metabolically based intervention, the ketogenic diet may serve as a safe and complementary approach to conventional pharmacological treatments, addressing core pathophysiological mechanisms underlying bipolar disorder and potentially other mood disorders.

## 5. Metabolic Dysfunction in Alzheimer’s Disease: Implications for Ketogenic Therapy

Alzheimer’s disease (AD) is a progressive neurodegenerative disorder characterized by a decline in cognitive function, most notably progressive impairment of memory, and represents one of the most common causes of dementia. Dementia affects an estimated 40 million people worldwide, primarily individuals over the age of 60 [121], and AD is currently the seventh leading cause of death in the United States [122]. Alzheimer’s disease is classified by age of onset. Individuals who develop symptoms after the age of 65 are classified as having late-onset AD, whereas those who develop symptoms before age 65 are diagnosed with early-onset AD. Patients with early-onset AD often present with atypical clinical features, which can delay diagnosis and are frequently associated with a more aggressive disease course [123]. The progression of AD involves distinct stages of cognitive decline and functional impairment, including a preclinical (or presymptomatic) stage, mild cognitive impairment, and dementia. Memory impairment is typically the first noticeable symptom of AD. Individuals with mild impairment experience greater memory difficulties than expected for their age; however, these deficits do not yet significantly interfere with daily functioning. Mild impairment is often characterized by episodic short-term memory loss, with difficulty retaining new information while largely preserving long-term memories [124]. As the disease progresses to dementia, cognitive and functional impairments become more pronounced. Dementia due to Alzheimer’s disease is further categorized into mild, moderate, and severe stages based on the extent of cognitive decline and loss of independence [125].

Although several other forms of dementia exist, including Lewy body dementia and vascular dementia, Alzheimer’s disease is distinguished by its characteristic pathophysiology involving abnormal accumulation of amyloid-β (Aβ) peptide plaques and neurofibrillary tangles composed of tau protein [122,126]. Amyloid-β plaques are primarily composed of two peptide species, Aβ40 and Aβ42, which are normal metabolic byproducts generated from the cleavage of amyloid precursor protein (APP) by β-secretase and γ-secretase enzymes [127]. Although Aβ40 and Aβ42 are produced under normal physiological conditions, Alzheimer’s disease is characterized by excessive accumulation and deposition of these peptides, particularly the more aggregation-prone Aβ42. In addition to amyloid pathology, the disease is marked by the presence of neurofibrillary tangles (NFTs), which consist of abnormally hyperphosphorylated tau protein. Under pathological conditions, tau proteins detach from microtubules, misfold, and aggregate into paired helical filaments that accumulate within neuronal cell bodies (perikarya), axons, and dendrites. This accumulation disrupts microtubule integrity and impairs cytoskeletal structure, ultimately leading to neuronal dysfunction and degeneration.

The formation of NFTs is generally described as a three-stage process. The initial pre-tangle stage involves the accumulation of tau within the somatodendritic compartment of neurons. This is followed by the formation of mature neurofibrillary tangles, in which tau filaments are fully aggregated and displace the neuronal nucleus toward the periphery of the soma. The final stage consists of extracellular or “ghost” tangles, which remain after neuronal death. Because these tau aggregates are resistant to proteolytic degradation, they persist extracellularly as remnants of lost neurons [128,129]. In addition to these molecular hallmarks, individuals with Alzheimer’s disease exhibit pronounced structural brain changes, including cortical and hippocampal atrophy accompanied by ventricular enlargement, reflecting widespread neuronal loss [130].

### 5.1. Molecular Risk Factors and Neurotransmitter Dysregulation

A key pathogenic feature of Alzheimer’s disease is chronic neuroinflammation. Among the many proposed drivers of brain inflammation in AD, the most prominent are the disease’s defining pathological hallmarks: amyloid-β (Aβ) plaques and neurofibrillary tangles (NFTs). In AD, Aβ is thought to activate microglia—the resident immune cells of the central nervous system—which normally function to clear cellular debris, including Aβ plaques, through phagocytosis. However, over time, microglia become progressively activated and enlarged, ultimately losing their ability to efficiently clear Aβ deposits [131]. Although the early immune response may initially contribute to Aβ clearance, prolonged microglial activation exacerbates AD pathology by promoting sustained neuroinflammation. This occurs through a detrimental feed-forward loop, in which chronic microglial activation enhances Aβ accumulation, further amplifying inflammatory signaling in the brain [132,133]. Importantly, while microglial phagocytic capacity declines over time, their ability to produce pro-inflammatory cytokines remains largely unaffected [132]. Chronically activated microglia continue to release reactive oxygen species, cytokines, and other inflammatory mediators, all of which contribute to a persistent inflammatory environment in AD [134].

Clinical and experimental studies provide further support for the role of inflammation in AD pathology. For example, individuals with a history of head trauma exhibit increased cerebral Aβ levels accompanied by elevated interleukin-1 (IL-1), which upregulates amyloid precursor protein (APP) expression and promotes further Aβ production [135]. Elevated IL-1β has also been shown to stimulate the synthesis of other cytokines, including interleukin-6 (IL-6), which in turn activates cyclin-dependent kinase 5 (CDK5). CDK5 activity contributes to tau hyperphosphorylation, a hallmark feature of AD pathology [136]. In addition, IL-6 has been implicated in regulating APP synthesis through its interactions with other molecular signaling partners [137]. Beyond IL-1 and IL-6, increased levels of tumor necrosis factor-α (TNF-α) and macrophage inflammatory protein-1α (MIP-1α) have been observed in AD microglia following incubation with Aβ. Notably, TNF-α-converting enzymes may further influence APP metabolism by modulating α-secretase-mediated processing of APP [138]. Furthermore, mitochondrial dysfunction has been shown to enhance the recruitment of pro-inflammatory cytokines such as IL-1β, while simultaneously activating inflammatory signaling pathways that amplify ROS generation [139]. Together, chronic neuroinflammation, oxidative stress arising from mitochondrial dysfunction, and impaired cellular energy metabolism synergistically promote neuronal death. These interconnected processes contribute to one of the most prominent pathological outcomes of Alzheimer’s disease: progressive atrophy of brain regions critical for memory and cognition, particularly the hippocampus.

Neurotransmitter dysfunction is another prominent pathophysiological feature of Alzheimer’s disease (AD). One of the most well-established changes is reduced cholinergic activity, resulting in a significant decline in acetylcholine levels. This reduction is primarily due to degeneration of cholinergic neurons, particularly in the hippocampus and cerebral cortex—regions critically involved in learning, memory, and cognitive function [140]. AD is also associated with dysregulation of several other neurotransmitter systems, including glutamate, serotonin, and dopamine, all of which deviate from their normal concentrations in the brain. Notably, damage to glutamatergic neurons is commonly observed in AD, leading to excessive glutamate release. Overactivation of glutamatergic signaling, particularly through NMDA receptors, can induce excitotoxicity, a process that contributes to neuronal injury and cognitive decline.

This glutamatergic dysfunction is closely linked to amyloid-β (Aβ) pathology. Accumulation of Aβ can exacerbate excitotoxic injury by binding to glutamate receptors, promoting excessive glutamate release, and enhancing NMDA receptor overstimulation [141]. Elevated NMDA receptor activation increases intracellular calcium influx, which triggers a cascade of damaging events, including activation of proteolytic enzymes, generation of reactive oxygen species, and mitochondrial dysfunction [142]. In addition to excitatory neurotransmission abnormalities, inhibitory signaling is also disrupted in AD. Reduced activity of glutamate decarboxylase leads to decreased GABA levels and further elevations in glutamate concentrations. This imbalance between excitatory and inhibitory neurotransmission exacerbates glutamatergic dysregulation and contributes to neuronal hyperexcitability and neurodegeneration in AD [143].

### 5.2. Altered Metabolism

Mitochondrial dysfunction and impaired respiratory chain activity have been consistently observed in the brains of patients with Alzheimer’s disease. Notably, mitochondrial dysfunction has been linked to altered processing and localization of amyloid precursor protein (APP). APP has been shown to localize to mitochondria, where it interacts with components of the mitochondrial protein import machinery, including the translocase of the outer mitochondrial membrane (TOMM40) and the translocase of the inner mitochondrial membrane (TIM23). Formation of this complex disrupts normal mitochondrial protein import, leading to mitochondrial dysfunction characterized by impaired ATP synthesis, altered mitochondrial membrane potential, and reduced cytochrome c oxidase (COX) activity, a key component of electron transport chain complex IV, where molecular oxygen is reduced to water [144].

Although the precise sources of oxidative stress in AD remain incompletely defined, altered mitochondrial localization of APP represents a potential contributing mechanism. When COX activity is diminished, incomplete oxygen reduction can occur, leading to the formation of reactive oxygen species such as superoxide radicals and pre-ROS molecules such as hydrogen peroxide, which may subsequently generate additional reactive intermediates. Consequently, APP-mediated disruption of mitochondrial function can shift redox homeostasis and promote oxidative stress.

In addition to alterations in COX activity, studies have reported reduced expression and activity of other electron transport chain components, including mitochondrial ATP synthase. In AD brains, ATP synthase dysfunction has been associated with the loss of the oligomycin-sensitivity conferring protein subunit [145] and decreased O-GlcNacylation of the ATP synthase α-subunit [146,147]. These molecular alterations further compromise mitochondrial energy production. Collectively, these disruptions in mitochondrial structure and function contribute significantly to the pathophysiology of Alzheimer’s disease by impairing cellular ATP generation and promoting the accumulation of oxidative byproducts, which exacerbate neuronal damage and accelerate neurodegeneration.

In addition to mitochondrial dysfunction, alterations in brain energy metabolism have also been observed in patients with Alzheimer’s disease. One of the most consistent findings is a reduction in cerebral glucose catabolism, which has been strongly associated with cognitive decline and functional impairment in AD. Hypometabolism is particularly evident in specific brain regions, including the frontal, temporal, and parietal lobes, as well as the hippocampus—a structure critical for learning and memory formation [148,149,150,151]. Postmortem studies further reveal reductions in glucose transporter expression, which impair glucose uptake required for cellular energy production. Notably, levels of glucose transporter 1 (GLUT1) [152] and glucose transporter 3 GLUT3 [153] are reduced in the hippocampus and cerebral cortex of individuals with AD. GLUT1 is primarily expressed in endothelial cells of the blood–brain barrier and facilitates glucose transport into the brain, whereas GLUT3 is predominantly expressed in neurons and is responsible for neuronal glucose uptake from the extracellular environment to support energy metabolism [154]. Importantly, decreased expression of GLUT1 and GLUT3 in AD brain tissue has been correlated with tau hyperphosphorylation and increased neurofibrillary tangle density, suggesting that impaired glucose transport and energy metabolism may directly contribute to tau pathology and disease progression [155].

### 5.3. Pharmacological Treatments and the Rationale for Ketogenic Therapy

Currently, there is no cure for Alzheimer’s disease, and available treatments primarily aim to alleviate symptoms and slow disease progression. One of the most commonly prescribed classes of medications includes cholinesterase inhibitors, such as donepezil, rivastigmine, and galantamine. These drugs inhibit acetylcholinesterase (AChE), the enzyme responsible for degrading acetylcholine (ACh) in synaptic clefts. By inhibiting AChE, cholinesterase inhibitors increase synaptic ACh availability, thereby enhancing cholinergic receptor activation to partially compensate for the acetylcholine deficit observed in AD. Another therapeutic class includes NMDA receptor antagonists, such as memantine, which reduce excessive NMDA receptor activation to limit excitotoxicity and neuronal cell death [128]. Despite their widespread use, these pharmacological approaches have important limitations. Cholinesterase inhibitors provide only temporary symptomatic relief and are associated with adverse effects; for example, donepezil commonly causes nausea, diarrhea, vomiting, and muscle cramps [156]. Similarly, although NMDA receptor antagonists can be beneficial in some patients, they may produce side effects and are often less effective in individuals with mild Alzheimer’s disease [157].

Clinical trials evaluating high-affinity NMDA receptor antagonists have largely failed because of poor efficacy and intolerable side effects. One major limitation is that NMDA receptors exist in multiple subtypes, some of which are involved in neuroprotective signaling, while others activate pathways that promote neuronal damage. Currently available NMDA antagonists are not subtype-specific and therefore indiscriminately block all NMDA receptor subtypes, potentially disrupting normal neuroprotective processes and healthy neuronal signaling. Additionally, emerging research suggests that certain NMDA receptor populations exhibit reduced sensitivity to specific antagonists, further limiting therapeutic efficacy [158]. Given these challenges and limitations of existing pharmacological treatments, increasing attention has turned toward alternative and adjunctive approaches—including the potential therapeutic role of the ketogenic diet.

Consistent with mechanisms outlined in Section 2, ketone metabolism supports mitochondrial efficiency and redox stability in AD. By bypassing glycolysis and reducing reliance on glucose transporters (which are downregulated in AD), the increased β-hydroxybutyrate reduces reactive oxygen species production at mitochondrial complex I. Furthermore, ketone body metabolism increases the expression of mitochondrial uncoupling proteins, which enhance the efficiency of oxidative phosphorylation. These uncoupling proteins lower the mitochondrial membrane potential by allowing protons to re-enter the mitochondrial matrix, thereby reducing electron leakage from the electron transport chain and limiting ROS formation [41]. The increase in the NAD^+^/NADH ratio associated with ketogenic metabolism may protect mitochondria from oxidative damage by promoting more efficient electron transfer during oxidative phosphorylation [159,160]. While glycolysis reduces two molecules of NAD^+^ to NADH per glucose in the cytosol, oxidation of β-hydroxybutyrate reduces NAD^+^ within the mitochondrial matrix, shifting redox reactions to the mitochondria and potentially supporting mitochondrial metabolic efficiency.

Most importantly, the ketogenic diet has been shown to alleviate neuroinflammation in the brain [161]. This effect may be particularly notable in Alzheimer’s disease, as chronic neuroinflammation may lead to progressive neurodegeneration and neuronal dysfunction. Because inflammation is a complex and multifactorial process, the precise mechanisms underlying this effect remain incompletely understood. However, several hypotheses have been proposed. One such mechanism suggests that β-hydroxybutyrate acts as a signaling molecule that inhibits class I histone deacetylases. HDAC activity has been linked to reduced expression of brain-derived neurotrophic factor, which can promote microglial and astrocytic activation and thereby exacerbate neuroinflammatory responses. Accordingly, inhibition of HDACs by β-OHB may suppress neuroinflammation [161,162,163]. In addition, β-OHB has been shown to inhibit activation of the nuclear factor kappa B (NF-κB) signaling pathway, a central mediator of the inflammasome [7]. In support of this mechanism, preclinical studies in diabetic mice demonstrated that β-OH treatment reduces inflammation by lowering nLRP3 inflammasome activity and pro-inflammatory cytokine levels, including IL-1β and TNF-α [164]. In another study, administration of a ketogenic diet (6.3:1 ratio of fats to carbohydrate plus protein) reduced brain inflammation in mice with autoimmune encephalomyelitis characterized by elevated inflammatory cytokine and chemokine expression, including IL-1β, IL-6, and TNF-α [165]. KD may also influence inflammatory responses through ketone body-mediated microRNA (miRNA) levels. A recent review suggests that KD-miRNA interactions may affect neurodegenerative disease processes via pathways related to BDNF, inflammation, and cognitive function [35]. For example, in pediatric patients with autism, KD significantly reduced miR-134-5p and miR-132-3p levels, while increasing miR-375-3p [166]. Changes in microRNA expression, including miR-134, miR-132, and miR-34a, have been associated with neurodevelopmental and cognitive functions [35].

As discussed previously, microglial activation contributes substantially to neuronal damage through the release of neurotoxic factors such as reactive oxygen species and pro-inflammatory cytokines. One study demonstrated that β-OHB significantly reduced lipopolysaccharide (LPS)-induced expression of pro-inflammatory cytokines and inflammatory enzymes in microglial cells. This anti-inflammatory effect was primarily attributed to suppression of NF-κB signaling, as β-OHB prevented degradation of IκB-α and inhibited nuclear translocation of the NF-κB p65 subunit, both of which are required for NF-κB activation. Collectively, these findings highlight β-OHB’s potential to modulate neuroinflammatory signaling pathways and attenuate inflammation-related neuronal damage [167].

In addition, several studies have reported that a high-glycemic diet is associated with increased cerebral amyloid burden [168], suggesting that limiting glycemic intake may help reduce amyloid accumulation. Furthermore, clinical trials have demonstrated that administration of ketone supplements or medium-chain fatty acids improves cognitive performance in patients with mild to moderate Alzheimer’s disease [169,170]. Together, these findings support growing evidence that metabolic interventions—particularly the ketogenic diet—may offer a promising therapeutic approach to Alzheimer’s disease. By improving mitochondrial function, reducing oxidative stress, and mitigating neuroinflammation, such interventions may help target key underlying pathophysiological mechanisms of the disease.

## 6. Conclusions

Researchers have explored the potential therapeutic benefits of the ketogenic diet (KD) in alleviating symptoms across several neurological disorders, including schizophrenia, bipolar disorder, and Alzheimer’s disease. The pathophysiology of these conditions reveals multiple overlapping features, most notably mitochondrial dysfunction and oxidative stress. By targeting shared metabolic vulnerabilities across disorders, ketogenic interventions may complement conventional therapies (Table 1).

Moreover, it is important to acknowledge that the pathophysiological mechanisms observed in schizophrenia, bipolar disorder, and Alzheimer’s disease overlap with those of other psychiatric and neurological conditions. As discussed, mitochondrial dysfunction and oxidative stress are shared features across these disorders. In addition, depression and anxiety—conditions that share several neurological characteristics with bipolar disorder—also involve disruptions in neurotransmitter systems and altered brain function. Parkinson’s disease similarly exhibits mitochondrial dysfunction and oxidative stress and shares key pathological features with Alzheimer’s disease, including neuroinflammation. These shared mechanisms suggest that therapeutic strategies targeting mitochondrial function, oxidative stress, and inflammation may have potential benefits across a broader spectrum of neuropsychiatric and neurodegenerative disorders.

Metabolic dysfunction in neurodegenerative disease may be linked to alterations in RNA processing, providing an additional mechanistic layer connecting cellular energy deficits to neuronal dysfunction. Changes in mitochondrial activity and substrate utilization can influence transcriptional and post-transcriptional regulation through redox-sensitive signaling and epigenetic mechanisms. Metabolites such as NAD^+^ and acetyl-CoA regulate chromatin-modifying enzymes and RNA-binding proteins, thereby shaping gene expression and alternative splicing. Recent studies have identified widespread splicing abnormalities in genes involved in synaptic function, mitochondrial homeostasis, and proteostasis [171,172]. This metabolic–transcriptomic interface may also help explain the effects of ketogenic interventions, as ketone bodies such as β-hydroxybutyrate act not only as energy substrates but also as signaling molecules that influence transcriptional programs, with potential downstream effects on RNA processing. Integrating transcriptomic perspectives with biochemical and metabolic models therefore provides a more unified view of neurodegenerative mechanisms, linking mitochondrial dysfunction and redox imbalance to gene expression and splicing dysregulation, and may help explain how metabolic therapies modulate both cellular energetics and RNA regulatory networks.

The classical ketogenic diet typically employs a 4:1 or 3:1 fat to combined protein and carbohydrate ratio, robustly inducing and maintaining ketosis. More moderate formulations, including 2:1 ratios, can still produce measurable ketosis—particularly in adults—though with somewhat lower ketone levels. Less restrictive variants such as the modified Atkins diet and low-glycemic index treatments do not adhere to strict ratios but can still achieve therapeutic ketosis depending on carbohydrate restriction and individual metabolic response; for example, the modified Atkins diet generally allows higher protein intake and approximates a 1:1 ratio of carbohydrates to protein while emphasizing increased fat consumption. Across these formulations, the ketogenic diet has the potential to target underlying pathophysiological mechanisms common to multiple psychiatric and neurological disorders, including mitochondrial dysfunction and oxidative stress. The restrictive macronutrient composition of the KD can limit dietary flexibility, complicate social eating, and require ongoing monitoring and support. These factors may be especially relevant in psychiatric populations, where cognitive, motivational, and socioeconomic barriers can further impact long-term compliance.

Additionally, the emerging role of the gut microbiome in mental health and neurodegenerative disease underscores the importance of integrated therapeutic strategies that address both metabolic regulation and microbiome–brain interactions. Together, these considerations highlight the potential value of dietary interventions that take into account systemic metabolic, inflammatory, and microbiota-related influences on brain function.

Another important consideration is that individuals with major mental illnesses—particularly bipolar disorder—have an elevated risk of developing type 2 diabetes mellitus [173,174,175]. In the context of Alzheimer’s disease, emerging evidence has introduced the concept of type 3 diabetes mellitus (T3DM), in which brain insulin resistance contributes to disease etiology. Both preclinical and clinical studies have shown beneficial effects of antidiabetic and insulin-sensitizing medications on multiple aspects of Alzheimer’s disease pathology, including potential improvements in amyloid-β clearance. These findings support the hypothesis that insulin resistance plays a role in the development and progression of AD [176,177,178]. Despite this, it is important to consider the translational gap between preclinical and clinical studies when evaluating the therapeutic potential of KD. While rodent models provide insights into mechanisms by which the ketogenic diet may mitigate pathophysiology, they do not capture the complexity of human psychiatric and neurodegenerative disorders.

Although further clinical trials are needed to fully establish the therapeutic potential of the ketogenic diet, existing preclinical and clinical evidence suggest that KD may represent a promising alternative or adjunctive treatment for complex psychiatric and neurodegenerative disorders. The ketogenic diet may be particularly beneficial for patients who have shown limited response to conventional pharmacological therapies or could be used in combination with existing treatments to enhance therapeutic efficacy—an area that warrants continued investigation.

## Figures and Tables

**Table 1:** Summary of Key Biochemical Alterations in the Pathophysiology of Schizophrenia, Bipolar Disorder, and Alzheimer’s Disease.

Disorder	Chief Biochemical Alterations
Schizophrenia	Neurotransmitter dysregulation: dopamine and glutamate Oxidative stress Energy metabolism dysfunction, including mitochondrial and glycolytic impairments
Bipolar Disorder	Mitochondrial dysfunction Oxidative stress Neurotransmitter dysregulation: dopamine, glutamate, GABA
Alzheimer’s Disease	Mitochondrial dysfunction Oxidative stress Glucose hypometabolism Neuroinflammation Neurotransmitter dysregulation: acetylcholine, glutamate, GABA

## Data Availability

No new data were created or analyzed in this study. Data sharing is not applicable to this article.

## Abbreviations

The following abbreviations are used in this manuscript:

Aβ	amyloid β-peptide
ACh	acetylcholine
AChE	acetylcholinesterase
AD	Alzheimer’s disease
APP	amyloid precursor protein
ATP	adenosine triphosphate
BBB	blood–brain barrier
BD	bipolar disorder
BDNF	brain-derived neurotrophic factor
b-OHB	beta-hydroxybutyrate
CDK5	cyclin-dependent kinase 5
CNS	central nervous system
COX	cytochrome oxidase
DAG	diacylglycerol
D2	dopamine-2
DGKH	diacylglycerol kinase eta
DSM-5	Diagnostic and Statistical Manual of Mental Disorders, 5th edition
GABA	Gamma aminobutyric acid
GLUT	glucose transporter
GSH	glutathione
GWAS	genome-wide association study
HDAC	histone deacetylase
IL-1	interleukin 1
IL-6	interleukin 6
KD	ketogenic diet
LPS	lipopolysaccharide
MCT	monocarboxylate transporter
MRI	magnetic resonance imaging
mTOR	mammalian target of rapamycin
NADH	Nicotinamide adenine dinucleotide
NADPH	Nicotinamide adenine dinucleotide phosphate
NDUFV2	NADH dehydrogenase flavoprotein 2
NFT	Neurofibrillary tangle
NF-κB	Nuclear factor kappa B
NO	Nitric oxide
NMDA	N-methyl-D-aspartate
PKC	protein kinase C
RNS	reactive nitrogen species
ROS	reactive oxygen species
SNP	single nucleotide polymorphism
TNF	tumor necrosis factor
